# Supplementary material for: Lenvatinib or Sorafenib Treatment Causing a Decrease in Skeletal Muscle Mass, an Independent Prognostic Factor in Hepatocellular Carcinoma: A Survival Analysis Using Time-Varying Covariates
Source: Cancers (Basel). 2023 Aug 23;15(17):4223. doi: 10.3390/cancers15174223 (PMC10486953; doi:10.3390/cancers15174223)
Supplement: Supplementary file 1 [file cancers-15-04223-s001.zip › cancers-2560507-supplementary.pptx]

## Slide 1
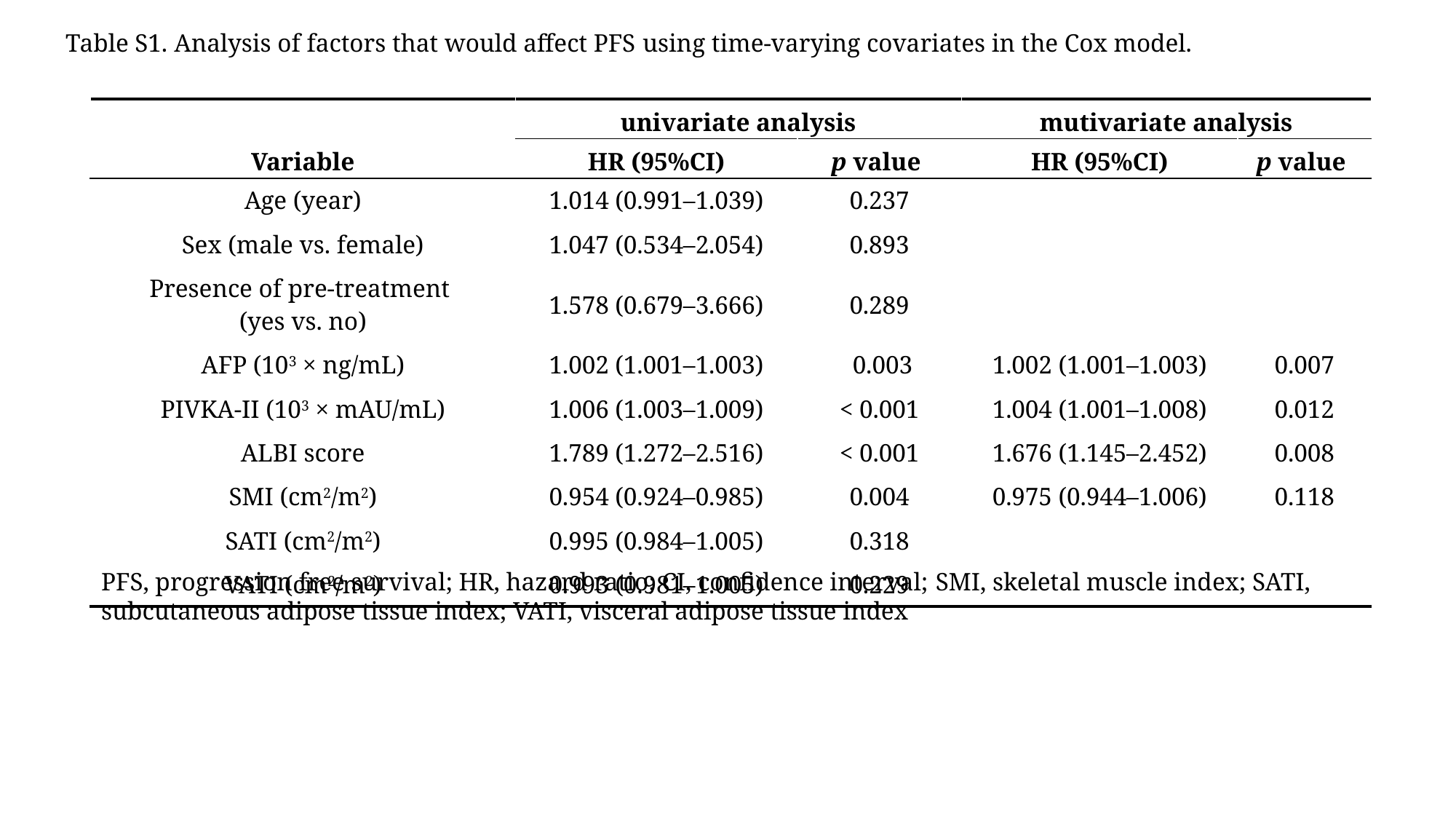

Table S1. Analysis of factors that would affect PFS using time-varying covariates in the Cox model.
| | univariate analysis | | mutivariate analysis | |
| --- | --- | --- | --- | --- |
| Variable | HR (95%CI) | p value | HR (95%CI) | p value |
| Age (year) | 1.014 (0.991–1.039) | 0.237 | | |
| Sex (male vs. female) | 1.047 (0.534–2.054) | 0.893 | | |
| Presence of pre-treatment (yes vs. no) | 1.578 (0.679–3.666) | 0.289 | | |
| AFP (103 × ng/mL) | 1.002 (1.001–1.003) | 0.003 | 1.002 (1.001–1.003) | 0.007 |
| PIVKA-II (103 × mAU/mL) | 1.006 (1.003–1.009) | < 0.001 | 1.004 (1.001–1.008) | 0.012 |
| ALBI score | 1.789 (1.272–2.516) | < 0.001 | 1.676 (1.145–2.452) | 0.008 |
| SMI (cm2/m2) | 0.954 (0.924–0.985) | 0.004 | 0.975 (0.944–1.006) | 0.118 |
| SATI (cm2/m2) | 0.995 (0.984–1.005) | 0.318 | | |
| VATI (cm2/m2) | 0.993 (0.981–1.005) | 0.229 | | |
PFS, progression free survival; HR, hazard ratio; CI, confidence interval; SMI, skeletal muscle index; SATI, subcutaneous adipose tissue index; VATI, visceral adipose tissue index

## Slide 2
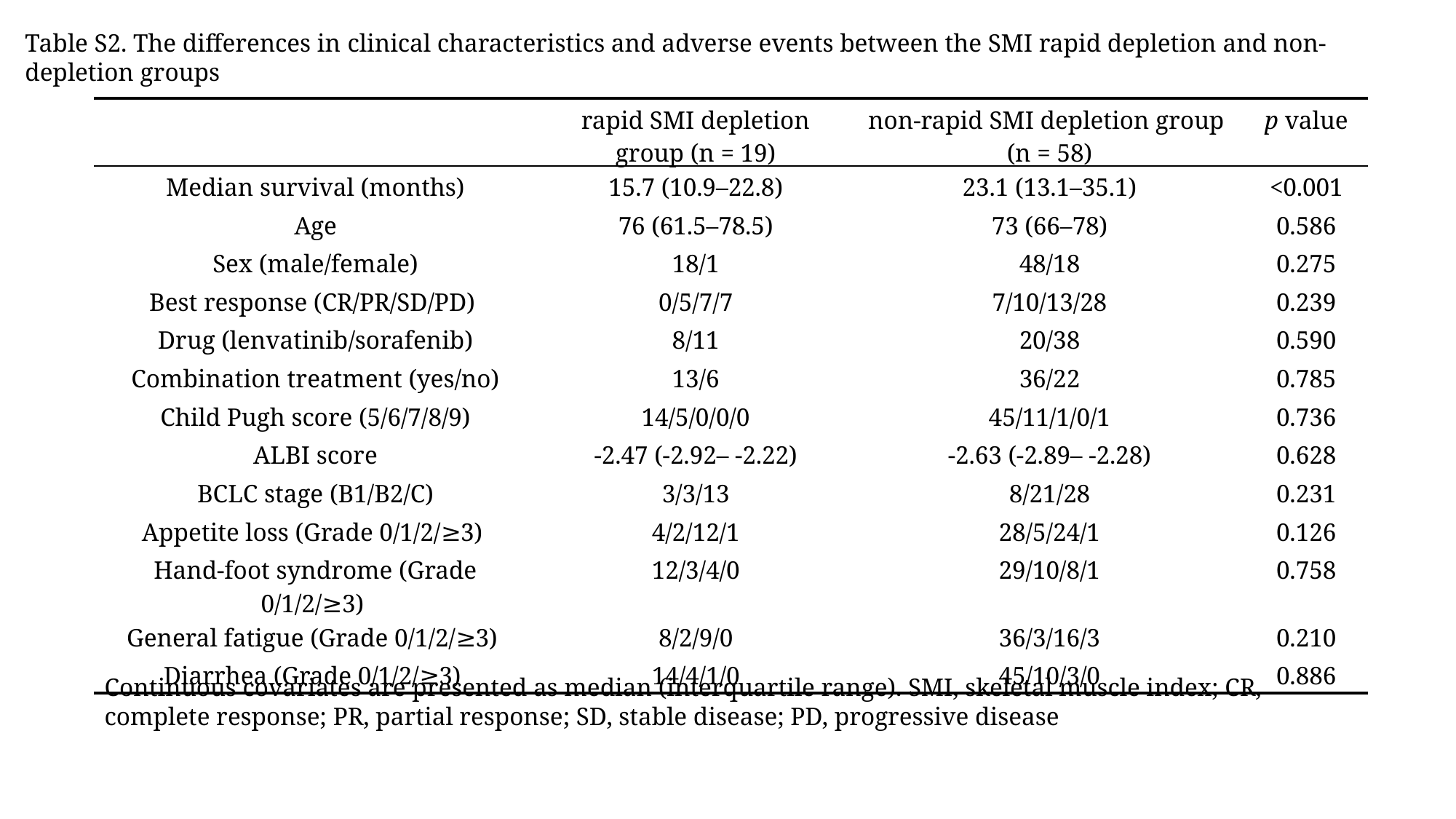

Table S2. The differences in clinical characteristics and adverse events between the SMI rapid depletion and non-depletion groups
| | rapid SMI depletion group (n = 19) | non-rapid SMI depletion group (n = 58) | p value |
| --- | --- | --- | --- |
| Median survival (months) | 15.7 (10.9–22.8) | 23.1 (13.1–35.1) | <0.001 |
| Age | 76 (61.5–78.5) | 73 (66–78) | 0.586 |
| Sex (male/female) | 18/1 | 48/18 | 0.275 |
| Best response (CR/PR/SD/PD) | 0/5/7/7 | 7/10/13/28 | 0.239 |
| Drug (lenvatinib/sorafenib) | 8/11 | 20/38 | 0.590 |
| Combination treatment (yes/no) | 13/6 | 36/22 | 0.785 |
| Child Pugh score (5/6/7/8/9) | 14/5/0/0/0 | 45/11/1/0/1 | 0.736 |
| ALBI score | -2.47 (-2.92– -2.22) | -2.63 (-2.89– -2.28) | 0.628 |
| BCLC stage (B1/B2/C) | 3/3/13 | 8/21/28 | 0.231 |
| Appetite loss (Grade 0/1/2/≥3) | 4/2/12/1 | 28/5/24/1 | 0.126 |
| Hand-foot syndrome (Grade 0/1/2/≥3) | 12/3/4/0 | 29/10/8/1 | 0.758 |
| General fatigue (Grade 0/1/2/≥3) | 8/2/9/0 | 36/3/16/3 | 0.210 |
| Diarrhea (Grade 0/1/2/≥3) | 14/4/1/0 | 45/10/3/0 | 0.886 |
Continuous covariates are presented as median (interquartile range). SMI, skeletal muscle index; CR, complete response; PR, partial response; SD, stable disease; PD, progressive disease
